# Supplementary material for: Incidence of Acute Kidney Injury in Polytrauma Patients and Predictive Performance of TIMP2 × IGFBP7 Biomarkers for Early Identification of Acute Kidney Injury
Source: Diagnostics (Basel). 2022 Oct 13;12(10):2481. doi: 10.3390/diagnostics12102481 (PMC9601128; doi:10.3390/diagnostics12102481)

Supplemental material

Supplemental Figure S1. Biological and Functional Markers

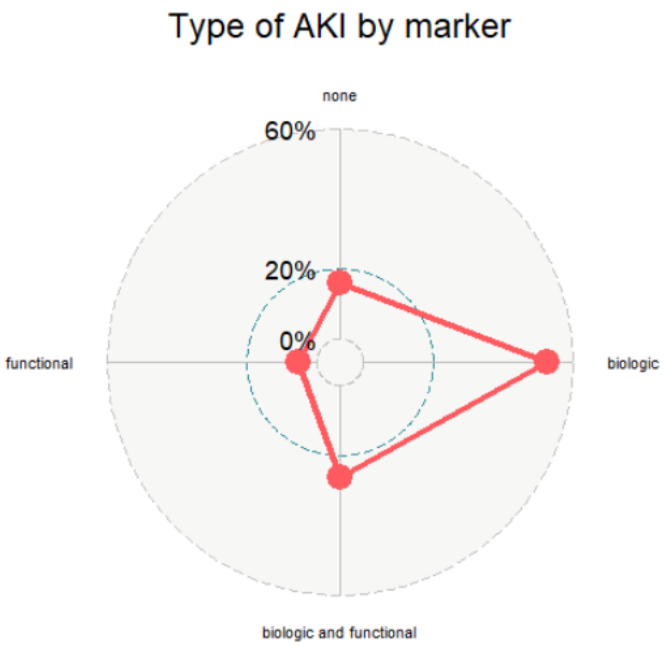

Supplemental Figure S2. Correlations Heat Maps

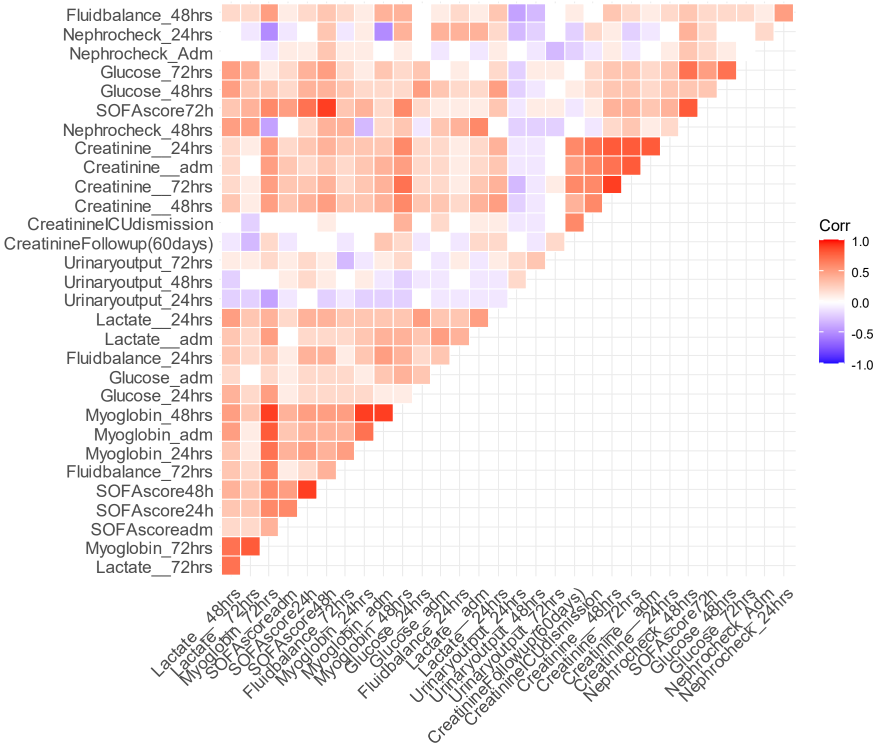

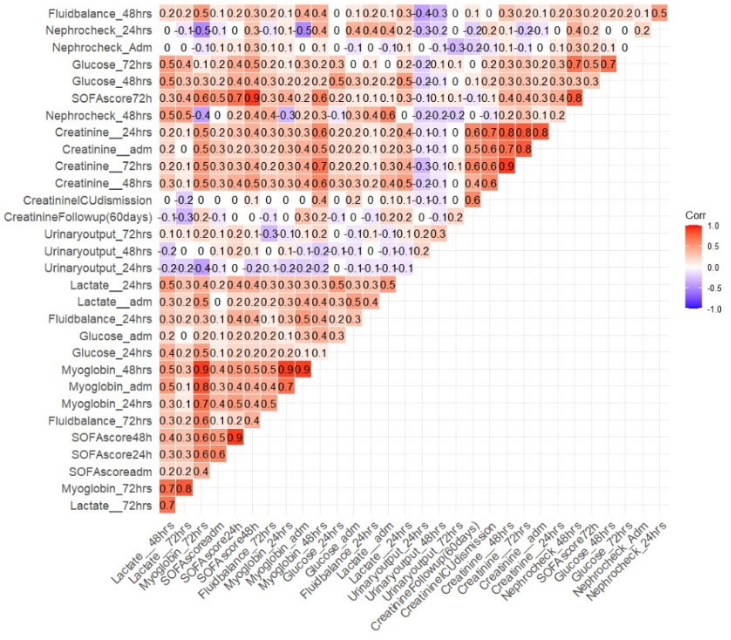

Association between continuous and categorical variables were tested through Wilcoxon signed-rank test.

Supplemental Figure S3. ROC analysis of multivariable model

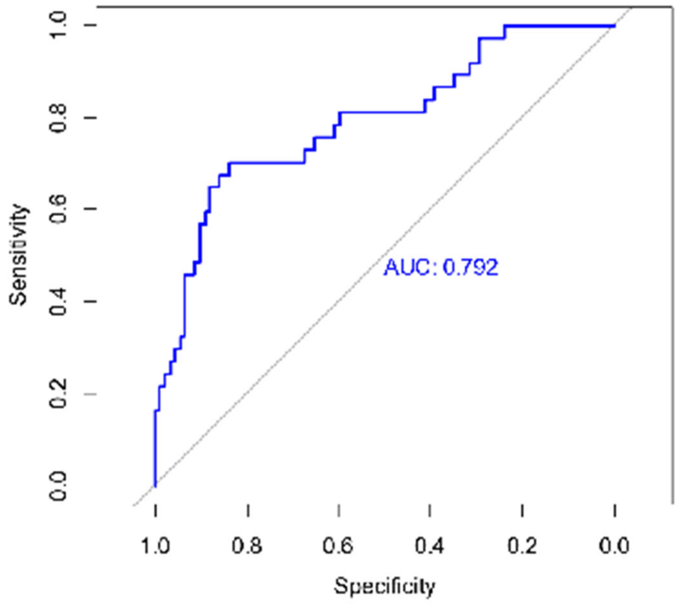

Supplement: Supplementary file 1 [file diagnostics-12-02481-s001.zip › Supplemental material.pdf]
