# Supplementary material for: Incidence of Acute Kidney Injury in Polytrauma Patients and Predictive Performance of TIMP2 × IGFBP7 Biomarkers for Early Identification of Acute Kidney Injury
Source: Diagnostics (Basel). 2022 Oct 13;12(10):2481. doi: 10.3390/diagnostics12102481 (PMC9601128; doi:10.3390/diagnostics12102481)
Supplement: Supplementary file 1 [file diagnostics-12-02481-s001.zip › Supplemental Tables Def.pdf]

Supplemental Tables

Supplemental Table S1. Univariate-multivariate analysis.

		Univariate			Multivariable		
**Characteristic**	N	OR[1]	95% CI[1]	p-value	OR[1]	95% CI[1]	p-value
**Age**	152	1.05	1.03, 1.07	<0.001	1.04	1.02, 1.07	**0.002**
**Gender**	152						
**Female**		—	—		—	—	
**Male**		1.93	0.73, 6.11	0.2	2.82	0.80, 12.3	0.13
**BMI**	152	1.08	0.99, 1.17	0.078			
**SBP**	152	0.99	0.98, 1.00	0.13			
**FC**	152	1.00	0.98, 1.01	0.8			
**Shock Index**	152	1.55	0.50, 4.71	0.4	2.40	0.57, 9.41	0.2
**ISS**	151	0.98	0.95, 1.02	0.4	0.97	0.92, 1.02	0.3
**GCS**	152	1.02	0.94, 1.12	0.6			
**Type of injury**	152						
**Blunt**		—	—		—	—	
**Penetrating**		1.79	0.50, 5.92	0.3			
**Injury Meccanism**	152						
**Bicycle hit**		—	—		—	—	
**Fall <6 m**		1.70	0.45, 7.29	0.4			
**Fall >6 m**		2.83	0.30, 23.8	0.3			
**Machinery**		1.42	0.06, 15.1	0.8			
**Motor vehicle traffic accident**		2.13	0.55, 9.27	0.3			
**Motorcycle**		2.02	0.57, 8.44	0.3			
**Other**		1.59	0.38, 7.24	0.5			
**Pedestrian**		2.32	0.54, 10.9	0.3			
**Type 2 diabetes**	152	1.90	0.59, 5.83	0.3			
**Hypertension**	152	4.43	2.07, 9.66	<0.001			

		Univariate			Multivariable		
**Characteristic**	N	OR[1]	95% CI[1]	p-value	OR[1]	95% CI[1]	p-value
**Anti-platelet and Anti-coagulation Therapy**	152	5.28	2.29, 12.6	<0.001			
**Previous cardiac disease**	152	6.12	2.20, 18.8	<0.001	3.75	1.05, 15.0	**0.048**
**Previous renal disease**	152	4.93	0.46, 108	0.2			
**[TIMP-2] × [IGFBP7] Admission***	132	1.43	0.99, 2.09	0.055	1.53	0.99, 2.40	0.055

[1]OR = Odds Ratio, CI = Confidence Interval.* For every unit increase in AKIrisk score.

Supplemental Table S2: Renal Outcomes according to **[TIMP-2] × [IGFBP7] values at admission**

**Characteristic**	**Entire Cohort= 153[1]**	**[TIMP-2] × [IGFBP7] Admission <=0.03, N = 50[1]**	**[TIMP-2] × [IGFBP7] Admission > 0.3 AKI, N = 82[1]**	**p-value[2]**
**sCr Admission**	0.84 (0.70, 1.01)	0.82 (0.70, 0.99)	0.87 (0.71, 1.08)	0.3
**sCr 24hrs**	0.82 (0.70, 0.99)	0.80 (0.72, 0.95)	0.86 (0.70, 1.05)	0.2
**sCr 48hrs**	0.81 (0.68, 1.07)	0.78 (0.68, 0.92)	0.84 (0.72, 1.09)	0.3
**sCr 72hrs**	0.75 (0.64, 0.92)	0.74 (0.64, 0.85)	0.74 (0.66, 0.92)	0.8
**Myoglobin Admission**	974 (458, 1,888)	964 (411, 2,888)	979 (518, 1,664)	0.8
**Myoglobin 24hrs**	1,354 (630, 2,705)	1,449 (662, 2,856)	1,362 (643, 2,741)	0.8
**Myoglobin 48hrs**	697 (258, 1,595)	774 (372, 1,076)	710 (211, 2,728)	0.7
**Myoglobin 72hrs**	835 (442, 2,236)	665 (442, 1,680)	893 (371, 1,366)	>0.9
**Glucose Admission**	150 (128, 179)	158 (131, 189)	148 (128, 177)	0.4

Characteristic	Entire Cohort= 153[1]	[TIMP-2] × [IGFBP7] Admission <=0.03, N = 50[1]	[TIMP-2] × [IGFBP7] Admission > 0.3 AKI, N = 82[1]	p-value[2]
**Glucose 24hrs**	135 (118, 160)	143 (115, 167)	135 (121, 161)	0.7
**Glucose 48hrs**	129 (114, 152)	130 (114, 149)	129 (118, 153)	0.8
**Glucose 72hrs**	129 (113, 150)	137 (114, 156)	127 (116, 146)	0.4
**PCT Admission**	0.27 (0.07, 1.15)	0.14 (0.06, 0.54)	0.34 (0.11, 0.94)	0.5
**PCT 24hrs**	0.87 (0.32, 3.05)	1.65 (0.14, 5.10)	0.62 (0.33, 2.81)	0.5
**PCT 48hrs**	0.9 (0.5, 3.9)	1.3 (0.8, 3.9)	1.6 (0.3, 4.6)	0.7
**PCT 72hrs**	0.7 (0.3, 2.1)	1.3 (0.8, 2.8)	0.4 (0.3, 2.1)	0.063
**FB 24hrs**	1,430 (457, 2,636)	1,441 (498, 3,100)	1,307 (446, 2,620)	0.8
**FB 48hrs**	850 (-16, 1,938)	727 (110, 1,823)	1,012 (61, 1,946)	0.7
**FB 72hrs**	860 (102, 1,671)	555 (104, 1,263)	1,021 (289, 2,004)	0.2
**% FO 24hrs**	18 (5, 35)	18 (8, 36)	16 (5, 35)	0.9
**% FO 48hrs**	11 (0, 25)	11 (1, 25)	13 (1, 25)	0.7
**% FO 72hrs**	11 (1, 20)	7 (1, 17)	14 (4, 24)	0.3
**UO 24hrs**	1,855 (1,289, 2,575)	1,910 (1,340, 2,950)	1,942 (1,322, 2,555)	0.7
**UO 48hrs**	2,300 (1,725, 3,000)	2,350 (1,725, 3,410)	2,110 (1,650, 2,850)	0.2
**UO 72hrs**	2,570 (1,888, 3,140)	2,775 (2,378, 3,442)	2,190 (1,770, 2,960)	**0.015**
**Furosemide 24hrs**	42 (28%)	12 (24%)	27 (33%)	0.3
**Furosemide 48hrs**	41 (36%)	16 (41%)	22 (37%)	0.7
**Furosemide 72hrs**	22 (26%)	9 (35%)	12 (24%)	0.4
**RRT 24hrs**	1 (0.7%)	0 (0%)	0 (0%)	>0.9
**RRT 48hrs**	4 (3.5%)	0 (0%)	3 (5.0%)	0.3
**RRT 72hrs**	4 (4.7%)	0 (0%)	3 (6.1%)	0.5

[1]Median (IQR); n (%). x[2]Wilcoxon rank sum test; Wilcoxon rank sum exact test. ACRONYM: FB, Fluid Balance; FO, Fluid Overload; PCT, procalcitonine; sCr, serum creatinine; RRT, renal replacement therapy; UO, urinary output;.
